# Statins Promote Long-Term Recovery after Ischemic Stroke by Reconnecting Noradrenergic Neuronal Circuitry

**DOI:** 10.1155/2015/585783

**Published:** 2015-09-10

**Authors:** Kyoung Joo Cho, So Young Cheon, Gyung Whan Kim

**Affiliations:** ^1^Department of Neurology, College of Medicine, Yonsei University, Seoul 120-752, Republic of Korea; ^2^Department of Anesthesiology and Pain, College of Medicine, Yonsei University, Seoul 120-752, Republic of Korea

## Abstract

Inhibitors of HMG-CoA reductase (statins), widely used to lower cholesterol in coronary heart and vascular disease, are effective drugs in reducing the risk of stroke and improving its outcome in the long term. After ischemic stroke, cardiac autonomic dysfunction and psychological problems are common complications related to deficits in the noradrenergic (NA) system. This study investigated the effects of statins on the recovery of NA neuron circuitry and its function after transient focal cerebral ischemia (tFCI). Using the wheat germ agglutinin (WGA) transgene technique combined with the recombinant adenoviral vector system, NA-specific neuronal pathways were labeled, and were identified in the locus coeruleus (LC), where NA neurons originate. NA circuitry in the atorvastatin-treated group recovered faster than in the vehicle-treated group. The damaged NA circuitry was partly reorganized with the gradual recovery of autonomic dysfunction and neurobehavioral deficit. Newly proliferated cells might contribute to reorganizing NA neurons and lead anatomic and functional recovery of NA neurons. Statins may be implicated to play facilitating roles in the recovery of the NA neuron and its function.

## 1. Introduction

Therapeutic aims after CNS injury are to prevent second complications in acute stage and to enhance functional recovery of the damaged brain in chronic stage. The neurological secondary implications contain urinary disorders, depression, or dementia [1]. Cardiac complications including sudden death are also frequent during recovery phase after stroke [2]. Response to increased serum cardiac enzyme and high blood pressure, plasma noradrenaline (NA) levels are often elevated [3, 4]. Dysfunction of the noradrenergic system is a frequent feature in various neurodegenerative disorders such as Alzheimer's disease and Parkinson's disease, as well as in acute ischemic stroke. At stroke onset, ischemic sites and lesion size may be related to different characteristics of autonomic dysfunction [5]. Malfunction in the noradrenergic system is also implicated in many psychiatric disorders manifesting abnormal social behavior, attention deficit, hyperactivity, anxiety, and depression. Brain ischemia induces pathological synaptic plasticity caused by delayed neuronal death and induces physiological plasticity which leads to structural reorganization resulting in functional recovery [6, 7]. NA has been reported to regulate neural stem cell and also act as a positive modulator for hippocampal neurogenesis in vitro and in vivo [8]. In adult mice, alteration of noradrenergic system could affect neurogenesis and consequently improve the performing ability to perceptual learning task [9].

Inhibitors of 3-hydroxy-3-methylglutaryl-coenzyme A (HMG-CoA) reductase are widely used for secondary stroke prevention [10]. Statin has been reported to have numerous effects on neuron survival, angiogenesis, and neurogenesis as well as lipid-lowering activity [11, 12]. Along with these therapeutic usage, it was been mainly investigated whether statin treatment, HMG-CoA reductase inhibition, could promote neurological recovery, perilesional, and contralesional neuronal plasticity in the post-acute stroke phase promotes [13]. Administration of rosuvastatin for 30 days after middle cerebral artery occlusion (MCAO) showed the effect on reducing dementia [13]. Similar results were also reported in treatment by simvastatin or atorvastatin [14]. Additionally, statin might be related to reducing risk of dementia [15–17], and it received attention regarding playing another role in neurodegenerative disease. In Parkinson's disease (PD) animal model, simvastatin treatment showed various neuroprotective effects by interacting with NMDA receptor [18] and also by regulating muscarinic M1/4 receptor [19]. Additionally, mevalonate pathway inhibited the neurites outgrowth by blocking the cell rounding rapidly induced [17].

However, it is extremely poorly reported that statin affects NA system which is deeply affected and altered by cerebral ischemic stroke and closely related to chronic disabilities of physical and emotional type. This current study presented the visualized noradrenergic neuronal circuitry using a genetic tracing method and investigated the effects of statins on the functional recovery after stroke by focusing on NA neuron circuitry and its function. We put the point on the behavioral improvement in chronic stage by statin treatment.

## 2. Materials and Methods

### 2.1. Animal Model and Drug Treatment

To obtain focal cerebral ischemia animal model, adult male C57BL/6 mice (23–26 g; Orient Co., Gyeonggi-do, South Korea) were housed in a 12 h light/dark cycle and permitted food and water. These mice were anesthetized by inhalation of isoflurane in N_2_O/O_2_ (%, 70 : 30) and subjected to fFCI by MCA occlusion with a surgical nylon suture (5.0, Silkam, B.Braum, PA, USA) for 1 hour. A Laser Doppler flowmeter (Transonic System Inc., New York, USA) probe placed directly on the skull surface over the territory of the MCA (1 mm posterior and 5 mm lateral to bregma) measured regional CBF before and after occlusion and immediately before sacrifice. Blood pressure was monitored by cannulating a femoral artery with Pressure Transducer (Harvard Apparatus, Inc., Holliston, MA, USA). All procedures were approved by the animal care committee at Yonsei University medical college. Statin (atorvastatin) was dissolved to concentration of 1 mg/mL in 5% methanol (methanol 10 *μ*L in saline 20 mL). The prepared statin solution was treated with intraperitoneal injection with 10 mg/kg from 1 day after MCAO.

### 2.2. MRI Analysis and Infarct Size Measurement

To obtain consecutive brain damage images, we performed MRI scans of mice. For MRI analysis, mice were put on a horizontal bore (400 mm) after anesthetizing with isoflurane by 4.7 Teslar MR scanner (Brucker Biospin, Billerica, MA, USA). Images were obtained using a T2-weighted fast spin echo sequence. Diffusion images were also acquired. Temperature was maintained and respiration was monitored throughout the entire scan. Mice recovered quickly following the scan with a 100% survival rate. Among the obtained MR images, 4 cuts were used in infarct size measurement and were combined. Each damage region was measured by ImageJ and represented by relative value with area of contralateral side versus damaged area of ipsilateral side.

### 2.3. Recombinant Adenoviral Construction and Viral Infection

The procedure for generation of recombinant adenoviruses was described and performed previously [20, 21]. The PRS-WGA adenovirus was friendly gifted from Dr. Huh, Kyung Hee University. A gene cassette containing the WGA gene downstream of the PRS2x8 promoter was inserted into the pAdTrack plasmid and finally into the pAdEasy1. Recombinant adenoviral DNA was cut with* Pac*1 and transfected into HEK293 cells using Lipofectamine (Invitrogen, CA, USA). Viruses were harvested 5 days after transfection from the transfected HEK293 cells and amplified on dishes. The viral particles were purified by cesium chloride density gradient ultracentrifugation, dialyzed, and tittered.

Animals were anesthetized and placed in a stereotaxic instrument. After incision of the skin, a small burr hole was made directly at LC (5.4 mm posterior, 1.0 mm lateral, and 3.8 mm deep to bregma) and the viral vector was unilaterally injected into the LC of ischemic hemisphere using a Hamilton syringe (Hamilton Co., Nevada, USA). A single injection of 0.2~0.3 *μ*L of the concentrated adenovirus suspension (about 2 × 10^13^ cfu/mL) was used in this study. The scalp was then sutured, and then the mouse was returned to standard housing. After 2 days of virus injection, mice were perfused with 4% formaldehyde and the brains were removed for histological analysis.

### 2.4. Immunohistochemistry

Fixed mouse brains were sectioned with a cryostat to obtain 40 *μ*m sections, respectively. The sections were pretreated for 20 min with 1% H_2_O_2_ in PBS containing 0.3% Triton X-100 for inactivation of endogenous peroxidase activity and permeabilization of cells. The sections were then incubated for 30 min with 5% normal rabbit serum in PBS to block nonspecific protein-binding sites and incubated with anti-WGA polyclonal antibody (1 : 2000, Vector Laboratory, CA, USA) and anti-BrdU monoclonal antibody (1 : 200, AbD Serotec, UK) in PBS containing 0.3% Triton X-100 with 2% normal rabbit serum overnight at 4°C and for 2 hours at room temperature. After washing, the sections were incubated with biotin-labeled anti-goat IgG (1 : 200, Vector Laboratory) followed by Vectastatin ABC elite kit (Vector Laboratory), TSA kit (Perkin Elmer, Boston, USA), and Vectastatin ABC elite kit again. Signals were visualized with Ni^2+^-intensified diaminobenzidine/peroxide reaction kit (Vector Laboratory). Specimens were observed with a microscope and computerized digital camera system (Olympus, Tokyo, Japan: Provis) and an image analysis system and program (Adobe Photoshop, San Jose, CA).

### 2.5. BrdU Labeling and Tissue Processing

Cell proliferation was measured by the incorporation of the thymidine analogue 5′-bromo-2-deoxyuridine (BrdU) that is incorporated into the DNA of dividing cells in immunohistochemically detectable quantities during the S phase of cell division. After MCAO, the animals were twice injected intraperitoneally with BrdU (Roche) (50 mg/kg at a concentration of 10 mg/mL in 0.9% NaCl) at 24 hours and 1 hour before sacrifice. For immunohistochemical detection of incorporated BrdU, double-stranded DNA was denatured to a single-stranded form suitable for immunohistochemical detection on sections. Sections were incubated in 50% formamide in standard sodium citrate at 65°C for 2 hours and treated further with 2 M HCl at 35°C for 30 min. After being rinsed for 10 min at room temperature in 0.1 M boric acid, the sections were washed with PBS and then incubated with 0.03% H_2_O_2_ in methanol for 5 min. The sections were incubated with a primary antibody against BrdU (1 : 1,000, Roche Diagnostics) at room temperature for 30 minutes, washed with PBS, and reacted with a FITC-conjugated secondary antibody (1 : 200, Jackson ImmunoResearch, PA, USA) for 30 min at room temperature. After washing, stained tissue samples were mounted using Vectashield mounting medium (Vector Lab, Burlingame, CA, USA).

### 2.6. Behavioral Assessment-Light/Dark Transition Test and Resident-Aggression-Intruder Test

#### 2.6.1. Light/Dark Transition Test

The test is based on the aversive nature of mice to light and on their spontaneous exploratory behavior in new environments [22]. The apparatus for the test consisted of a dark chamber and a bright chamber. There are gates to pass between two chambers and mice are allowed to move freely between the two chambers. The test is performed in the bright illuminated chamber and the number of entries into the dark chamber evaluated for 5 min. Besides the number of transitions, latent time in dark chamber was evaluated.

#### 2.6.2. Resident-Aggression-Intruder Test

Mice were habituated in the same cage for at least over 1 week. Aggression level was assessed with three sessions at three-day interval. Evaluating items were duration of aggressive behavior and the number of aggression. Duration of aggression indicates the sum of time that determine resident animal the duration to show active move action toward intruder, social exploration, lateral threat, or clinch attack. Latency to attack was expressed by measuring the time between the introduction of the intruder and the first clinch attack.

### 2.7. Data Analysis

The data were expressed as mean ± S.D. The statistical comparisons were performed by unpaired *t*-test and one-way ANOVA (StatView, SAS Institute, Inc., Cary, NC, USA). The significance between the groups was assigned at ^*∗*^ *p* < 0.05 and ^*∗∗*^ *p* < 0.001.

## 3. Results

### 3.1. Adenovirus-Mediated NA Neuron Expressing WGA in the Locus Coeruleus (LC)

**Figure 1:**
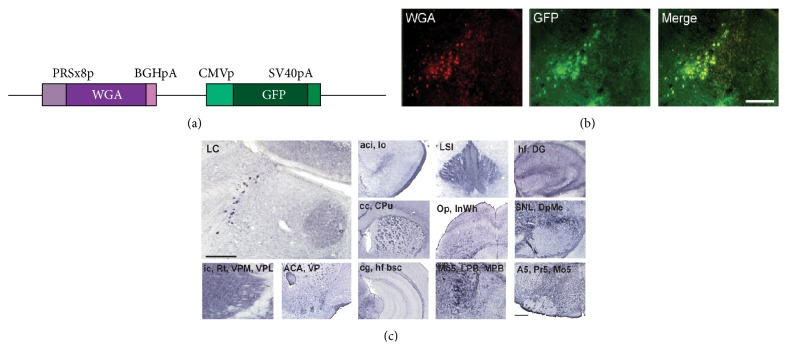
Expression of WGA and GFP in LC, detecting PRS-WGA adenovirus. (a) Schematic diagram representing the structure of the transgene. (b) WGA immunoreactivity in the noninjured mouse brain. After 2 days of PRS-WGA adenovirus injection in the LC, LC-originated WGA protein was detected in NA neurons and other areas. Scale bars = 500 *μ*m.

Adenoviral construct used in this study contains the wheat germ agglutinin (WGA) gene under control of the eight copies of PRS promoter and the green fluorescent protein (GFP) gene under the control of the cytomegalovirus (CMV) promoter, which makes it possible to detect viral delivery to the target sites (Figure 1(a)). After injection of the PRS-WGA adenovirus to the LC, localization of GFP expression and WGA protein was examined on adjacent coronal sections. Colocalization of GFP and WGA demonstrates that WGA protein was synthesized in NA neurons of the LC in our previous report [21]. In sagittal sections of noninjured brain, intense WGA immunoreactivity was detected in the pontine reticular nucleus, motor and sensory trigeminal nucleus, thalamic nucleus, lateral hypothalamic area, deep mesencephalic nucleus, superior colliculus, caudate putamen, corpus callosum, anterior commissure, and olfactory bulb (Figure 1(b)). WGA immunoreactivity was also observed in the parabrachial nucleus, A5 NA cells, substantia nigra, lateral habenular nucleus, hippocampus, optic tract, and piriform cortex in coronal sections (Figure 1(b)). In adenovirus infected mice there was no evidence of cell loss or tissue damage due to the viral infection in LC. All of the experimental animals that recovered after the infection remained healthy until being sacrificed without exhibiting any behavioral abnormalities.

### 3.2. Transsynaptic Transfer of WGA in Noradrenergic Neurons of Vehicle-Treated and Statin-Treated Mice after MCA Occlusion

**Figure 2:**
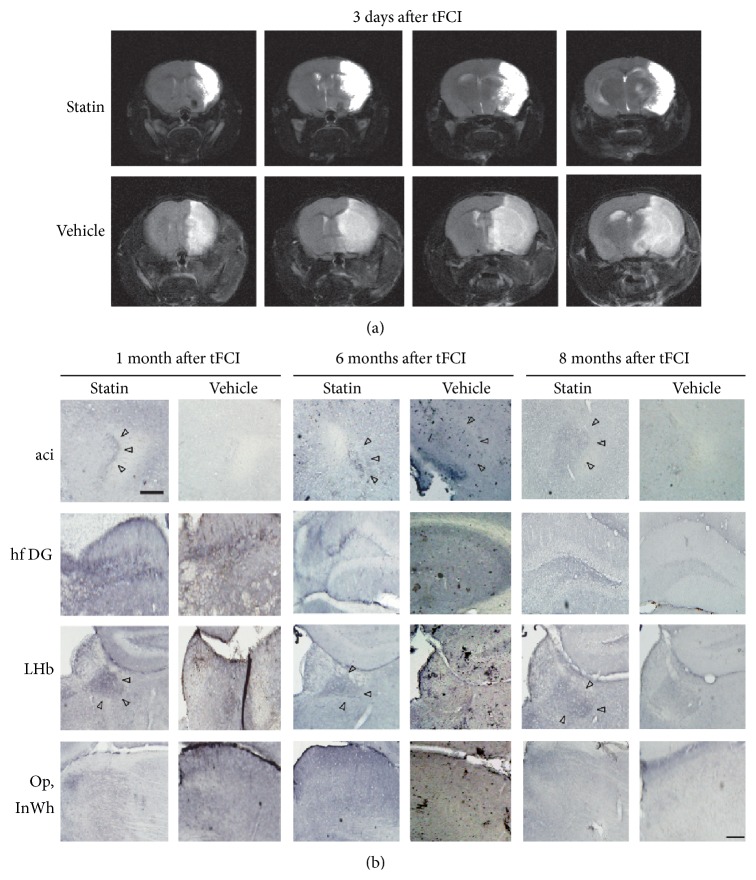
Immunoreactivity of WGA in the tFCI mouse brain. WGA immunoreactivity at 30, 180, and 240 days after tFCI in the vehicle- and atorvastatin-treated groups showed that NA circuitry in the atorvastatin-treated group recovered faster than in the vehicle-treated group at 180 days. aci: anterior commissure, intrabulbar part; hf DG: hippocampal fissure and dentate gyrus; LHb: lateral habenular nucleus; Op: optic nerve layer of the superior colliculus; InWh: intermediate gray layer of the superior colliculus. Scale bar = 500 *μ*m.

After transient focal cerebral ischemia (tFCI), WGA immunoreactivity showed the transsynaptic pattern of NA neurons according to day in each vehicle-treated or statin-treated mouse (Figure 2). NA circuits containing thalamic area, lateral hypothalamic area, cortex, and hippocampus were destroyed with severe infarction (data not shown). Although a large infarction was formed in the MCA territory, mice that survived slowly and gradually recovered the damaged area in the long-term stage (Figure 2(a)). When statin was given for a long period, the convalescence periods were faster than the vehicle-treated mice (Figure 2(b)). Whereas WGA immunoreactivity was not detected in some region such as superior colliculus from 1 month to 8 months in vehicle-treated mice after tFCI, a very strong WGA immunoreactivity was observed in the statin-treated mice in the intrabulbar part of the anterior commissure (aci) and lateral habenular nucleus (LHb). These features indicated that damaged NA circuits were partly reorganized over time in the area of previous lesion.

### 3.3. Ischemic Brain Damage and Convalescent Process after MCAO in Vehicle- or Statin-Treated Mice

**Figure 3:**
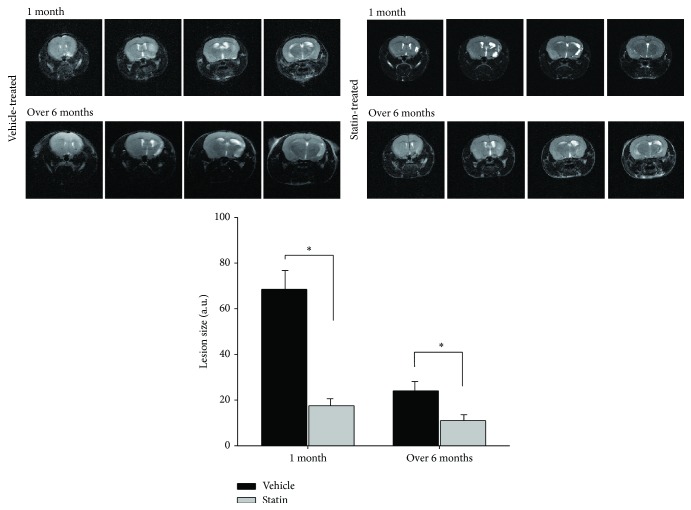
Ischemic brain damage and convalescent processes shown by magnetic resonance imaging (MRI) after MCAO.

Physiological data and regional cerebral blood flow (rCBF) were measured. There were no statistically significant differences in rCBF during ischemic condition between the group before occlusion and the group after reperfusion (10 min before occlusion, 100 ± 0%; 10 min after occlusion, 23.1 ± 5.3; 10 min after reperfusion, 96.2 ± 3.7; mean ± S.D. of each value; *n* = 9, each group). Presented in the magnetic resonance images (MRI), the infarcted area after reperfusion from MCAO was gradually reduced, and significantly statin-treated mice showed smaller infarct area than vehicle-treated mice (Figure 3). After a longer period, the damaged brain region was naturally ameliorated even in the vehicle-treated mice, but the recovery showed earlier in statin-treated mice than vehicle-treated mice. The differences in the recovery time determined the behavioral improvement and related to NA neuron circuitry, as shown in the result of the behavior test (Figure 5).

### 3.4. Neurogenesis after Ischemic Injury in Normal and Vehicle- or Atorvastatin-Treated Mice

**Figure 4:**
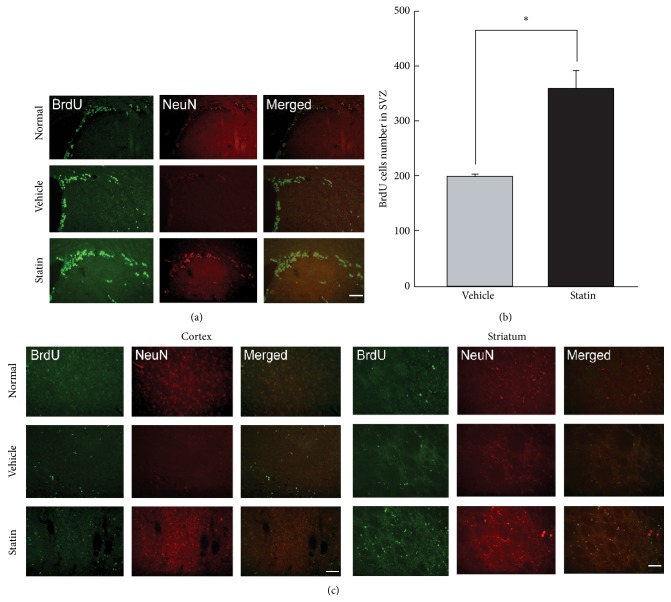
Neurogenesis after ischemic injury in normal and vehicle- or atorvastatin-treated group. (a) Cell proliferation in SVZ of the ischemic hemisphere is identified by BrdU (green) and NeuN (red) immunoreactivity. Many BrdU-positive cells are shown in the SVZ of both groups, with increased BrdU-positive cells in the striatum of the atorvastatin-treated group. (b) A quantitative graph representing the number of BrdU-positive cells, which is increased in the atorvastatin-treated group compared with the vehicle-treated group in the SVZ. (c) The migration of the proliferated cells is identified in the cortex and striatum each stained with BrdU (green) and NeuN (red). Scale bars = 20 *μ*m.

In postischemic brain injury, neuronal cells derived from neural precursor cells are newly born and the cells can be detected by bromodeoxyuridine (BrdU). The BrdU-immunopositive cells were observed in the postischemic cortex, striatum, and subventricular zone (SVZ) on the ipsilateral side of the ischemic infarct at 1 month and 6 months (Figures 4(a) and 4(b)). The BrdU-positive cells were more frequently observed in the ipsilateral side of the statin-treated mice than in the contralateral side. Especially at 1 month after tFCI, many BrdU- and NeuN-immunopositive cells were detected in the lesioned cortex and striatum at the direct site to ischemic damage by MCAO (Figure 4(c)). It could be implicated that newborn cells have arisen from the SVZ and migrated into striatum and even into cortical area. Moreover, the proliferation of neural cells is promoted by long-term statin treatment. At 6 months and 8 months after fFCI, the incidence of BrdU-positive cells in the ipsilateral striatum and cortex was significantly diminished compared to that of 1 month after fFCI (data not shown). The BrdU-positive cells were not detected in either striatum or cortex, but a few BrdU-positive cells were detected in SVZ and corpus callosum.

### 3.5. Neurological Outcome and Physiological Parameters according to NA Circuitry

**Figure 5:**
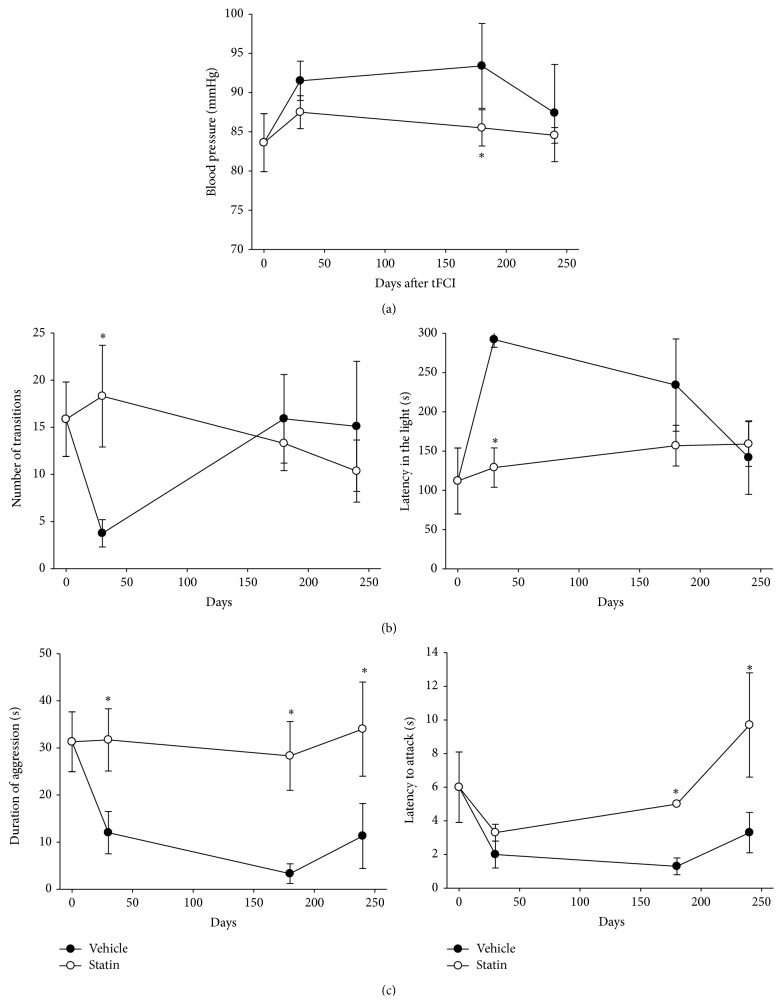
Physiological recovery and behavioral tests relating to the NA circuitry. (a) Profiles of blood pressure in vehicle-treated or atorvastatin-treated mice after tFCI. (b) Effects of atorvastatin on behavioral parameters in the light/dark test. The light/dark test was performed at 30, 180, and 240 days after tFCI in mice. The effect on mouse behavior in the light/dark test was determined by* p-test*. Data presented are the mean value ± S.E.M, ^*∗*^ *p* < 0.05 relative to the vehicle group. (c) Effects of atorvastatin on behavioral parameters in the aggression-intruder test in mice. Aggression-intruder test was performed at 30, 180, and 240 days after tFCI in mice. The effect on mouse behavior in the aggression-intruder test was determined by* p-test*. Data presented are mean value ± S.E.M, ^*∗*^ *p* < 0.05 relative to the vehicle group.

The mean arterial blood pressure (MABP) slightly increased until 4 months after fFCI and then gradually returned to the initial level at 8 months (Figure 5(a)). In contrast, statin treatment for a long period sustained the constant MABP level overall duration after tFCI. The body temperature and weight governed by NA system were also monitored according to month, but they just slightly oscillated with no significant changes. To evaluate the NA system alteration after tFCI and effects of statin on the NA system after tFCI, mice performed the light/dark transition test and aggression-intruder test. In the light/dark transition test, the number entering into the bright chamber from the dark chamber was convalescent at 6 months onward (Figure 5(b)). However, the duration of time spent in the light chamber for vehicle-treated mice was longer, over two times the normal, until 6 months and then it got back to normal at 8 months. Whereas the vehicle-treated mice had a fluctuation, the statin-treated mice were stable from the initial to final stage. The duration of aggression swung in the vehicle-treated mice; however, the duration in statin-treated mice did not vacillate (Figure 5(c)). The tendency to show aggression to new intruder was slightly higher in statin-treated mice than vehicle-treated mice in later stages (i.e., at 8 months). In general, statin-treated mice presented peaceful condition without upset through the observed duration.

## 4. Discussion

This study demonstrated that the NA neurons were transsynaptically labeled by the WGA-expressing adenoviral vector. By simply injecting the virus into the unilateral LC, the NA neural pathways were clearly visualized with great accuracy and high reproducibility from the LC to its projection areas of interest. The current study shows the following: (1) Transsynaptic tracing visualized NA circuitry during long-term recovery after ischemic damage and it is promoted by statin. (2) Eventually statin ameliorated the damaged behavioral function of NAergic neuron during long-term recovery period after stroke injury.

The WGA protein injection method had been used to visualize optic pathways in monkeys [23], olfactory systems in rodents [24], common afferent projections to the LC in rat [25], and connections of the A5 noradrenergic cell group in rat [26]. A genetic strategy employing cDNA for WGA as a transgene under the control of specific promoter elements was introduced in neural tracing study [27]. The present method successfully and reliably detected strong transsynaptically transferred WGA protein, which might be related to efficient infection of the adenovirus to the NA neurons, as well as the promoter elements (PRS promoter) being used for robust expression of WGA. PRS has previously been shown to be an NA-specific* cis* element binding to paired-like homeodomain factor Phox2a/Phox2b [28]. It has been confirmed that increasing copies of PRS cause synergistic activation of reporter gene expression, reaching maximal efficacy at eight copies [20], which is in agreement with successful visualization of the NA system in a mouse brain with a recombinant adenoviral vector expressing WGA under the control of PRS promoter elements in this study (Figure 1). NA is the neurotransmitter being implicated in many of these disorders and has been found to affect social behavior in both humans and animals [29, 30]. Therefore, investigating alterations of the NA systems in stroke may provide clues to the understanding of its symptomology, clinical courses, and adequate management. NA alteration may help to modulate the pathologically altered motor system in stroke patients, which resulted in upregulated coupling of damaged motor areas and consequently improved motor function [15]. Actually d-Amphetamine has a role in multiple brain transmitter systems, improved functional recovery after stroke, and its effect has been attributed to its noradrenergic systems [31]. Intense WGA immunoreactivity was initially detected in the most contralateral side and the ipsilateral nonlesioned areas, such as the thalamic nucleus, lateral habenular nucleus, and amygdala. These findings suggest that compensatory reinforcement of undamaged contralateral NA circuits occurred shortly after the onset of tFCI and then evolved into subsequent reorganization of NA circuitries in severely damaged area. Moreover, this process can be promoted by statin treatment. When NA synaptic activity increased pharmacologically, symptoms derived by cortical damage were recovered [32]. Synaptic activity also represents the functional recovery after a stroke in human. After focal cortical damage, circuitry reorganization is related to functional recovery, which takes place at the primary cortical level [33]. Therefore, investigating alterations of NA systems in stroke may provide clues to understand its symptomology, clinical courses, and adequate management. It has been known that an acute stroke induced by MCAO induced cellular proliferation or neurogenesis in the ipsilateral SVZ and that a large number of immature neurons migrate from the SVZ to ipsilateral infarcted areas at 2 weeks following insults [34, 35]. Statins are well-known drug to reduce cholesterol level. There are some reports that chronic statin treatment promotes endogenous neuronal cell proliferation, neurogenesis, and new synapse formation, as well as protecting the neuron from cell death in acute stage [12, 14]. In a long-term stage, we assume that statins are involved in facilitating long-term recovery after stroke by reconnecting the NA circuit using the newly proliferated cells in SVZ or subgranular zone (SGZ). BrdU immunohistochemistry, a thymidine analogue, was used at 1 month and 6 months after tFCI (Figure 4). It was also known that brain insults such as cerebral ischemia, causing neuronal death, are accompanied by increased neurogenesis in the SGZ and SVZ [36, 37]. BrdU-positive cells were observed in cortical and striatal lesions, and increased numbers of BrdU-positive cells were observed in SVZ and corpus callosum, which is consistent with the process of initial neuronal proliferation in SVZ and the subsequent migration into the ischemic lesions as a repair mechanism.

To assess the relationship of the remodeling process with functional recovery in the NA system, the alteration of blood pressure was measured starting 1 month after tFCI (Figure 5). Blood pressure and behavior change following acute ischemic stroke were related with the anatomical reorganization from 6-months after tFCI. Besides blood pressure [38, 39], dysfunction of the NA system has been widely known to be associated with body temperature [40, 41], body weight [42], and anxiety [29, 43]. Previous experiments indicate that blood pressure is significantly higher in mice having insular infarction [38, 39]. The insular cortex is directly connected with central nucleus of the amygdala and the posterior lateral hypothalamus [44, 45] belonging to NA system. Our results indicated that altered physiological dysfunction by acute ischemic stroke injury gradually recovered with the reorganization of damaged processes in the mature NA systems in those structures. The behavioral test using the light/dark test and the aggression-intruder test showed a significant difference between vehicle-treated mice and statin-treated mice (Figure 5). For longer period of up to 8 months after stroke attack, statin improved the malfunctioning anxiety and aggression from an earlier period. Vehicle-treated mice had altered anxiety-like behavior in a time-dependent manner following tFCI, as displayed by a significant increase in the time spent in the light chamber. In contrast to the fluctuating pattern related to the NA system in vehicle-treated mice, statin-treated mice were stable. Increase in NA signaling is associated with higher anxiety and decreased NA signaling with lower anxiety [46, 47]. It has been reported that oxidative stress in the CNS is related to anxiety [48, 49], and aggressive behavior is linked to oxidative stress, which is one of the main pathologies of ischemic stroke [50]. Our results also presented that after initial dysfunction of NA activities, gradual recovery followed along with reorganization of NA circuits in the infarcted structures.

## 5. Conclusion

Taken together, the present study visualized the NA circuitry of mice by tracing with the PRS-WGA adenoviral system after tFCI for a long duration. Our result showed the reorganization of damaged NA circuit after tFCI. In a chronic stage after stroke, statins were involved in long-term recovery of the NA circuitry and promoted the neurons to be proliferated and migrate to the infarcted area. Statins could ameliorate the NA-related malfunction after stroke in a chronic period.
